# Annotations

**Published:** 1911-06-03

**Authors:** 


### Annotations.

### Modern House Planning.

"The Hundred Best Houses" is the title of an attractive shilling guide to the exhibition of Town Planning which was opened this week by Mr. Burns at Gidea Park, near Romford. It contains plans and sketches of the 150 houses and cottages which have been erected on the estate by well-known architects in open competition, and it embodies an interesting collection of letters and expressions on the subject of the ideal house from both medical and architectural experts, to amateurs and faddists. The opinions on this question by such authorities as Mr. Thomas Hardy and Mr. Hall Caine (both of whom have had experience in the technical details of house building and construction), Mrs. Ayrton, Sir Frederick Treves, Miss Bentham Edwards, and Dr. John French are well worth consideration by those intending to plan a house: the remarks by the amateur hygienists are unconsciously amusing, since they reveal a primitive ignorance of the subject about which they presume to speak. The plans contained in the book are all interesting, some exceptionally so, and the exhibition is one of the most instructive to be seen at this season of exhibitions and shows. It remains merely to add that the profits from the sale of the guide book will go to the removal fund of King's College Hospital.

### The Latest Consumption Cure.

A good deal of attention has been aroused among the public by an alleged new cure for consumption which has been brought forward by Dr. A. de Szendeffy, of Budapest. The details given are, from a professional point of view, meagre in the extreme. The drug used is apparently a menthol-iodine-peptonate, rendered radioactive somehow or other; but how it is administered, and in what dosage, does not transpire. Whether this treatment is or is not likely to do good to sufferers from tuberculosis does not matter just for the moment. If those who claim that it does are reputable physicians, their claims must of course be carefully investigated; and should the new treatment turn out beneficial in any degree, the author of it will deserve the highest rewards. That, however, is for the future to show; and it will necessarily require months, or even years, of patient research by experts before the verdict can be finally reached. For the present there are two matters which may be usefully remembered. One is that during the last twenty years numbers of consumption cures have been belauded by their inventors, and in perfectly good faith. Many of those treatments have been the work of men of high standing in their respective countries, some of them scientific workers of the highest eminence, such as, for instance, the late Dr. Robert Koch. Yet we are still to-day without that which has been so toilfully sought—a magical and specific drug for combating the tubercle bacillus and its ravages. Therefore, in advocating caution, even scepticism, about iodine peptonate, we are merely taking to heart the lessons of the past. Yet equally must the opposite danger of hidebound prejudice and irrationalism be avoided. If the value of this preparation becomes clearly established, we must not let ourselves be blind to its virtue, but must welcome the new therapeutic weapon and add it at once to our armoury. The other matter has on previous occasions been made the subject of remark in The Hospital. The advertisement of Dr. Szendeffy's specific in articles which fill a column in a prominent position in the *Times* is not fair to the public or to the medical profession. Within a couple of hours of the appearance of the first one the present writer was asked by telephone to say whether he would recommend the treatment for a patient in Australia. And this incident is typical of what happens whenever the lay Press oversteps its proper function, as has been done in this instance.

### The Paper-bag Cookery.

Fomented by journalistic enterprise the latest newspaper boom has become a matter of very public interest. The popularising of this particular method of cooking food may be looked upon with favour by the medical profession as a hygienic advance. As a substitute for pots and pans the paper bag possesses obvious advantages as regards cleanliness and convenience. The rationale of enclosing viands in an air-tight envelope has long been appreciated by the best cooks, and this simple method of obtaining similar results deserves to become employed. The use of paper to wrap up meat preparatory to placing it in the oven is by no means new. The cleanliness of the paper-bag method is its recommendation, and the results achieved at recent demonstrations are encouraging. The method seems one particularly well adapted to small households, flats, and bachelor's "diggings." The details of the process, however, need careful attention, and the careless must not regard it as a means of turning fools into first-class chefs. Cooking "en papillote" certainly seems to be a valuable procedure in that it is able to improve the flavour of food by causing the retention of the empyreumatic substances in close contact with the food, and prevents to a large extent the usual smell of cooking from pervading the house and destroying the appetite.

### Doctors' Rural Telephones.

In connection with the reforms in the telephone service promised by Mr. Samuel, it is interesting to note that the concession made to farmers in scattered rural districts has also been thrown open to medical men. Many doctors, even if not actually living in a comparatively isolated village, have not infrequently small surgeries in remote hamlets which they visit at stated intervals, or once or twice a week. The facilities which are to be afforded in the shape of a single-line telephone connection for the sum of £3 a year will no doubt be welcomed, and will prove as great an advantage to the doctors as to the farmers. To the former it will mean the saving of unnecessary journeys and the possibility of receiving messages much earlier in the day—a matter frequently of great convenience and importance.

